# Electrochemical Testing of a New Polyimide Thin Film Electrode for Stimulation, Recording, and Monitoring of Brain Activity

**DOI:** 10.3390/mi13101798

**Published:** 2022-10-21

**Authors:** Samuel Ong, Aura Kullmann, Steve Mertens, Dave Rosa, Camilo A Diaz-Botia

**Affiliations:** NeuroOne Medical Technologies Corporation, Eden Prairie, MN 55344, USA

**Keywords:** implanted devices, neural recording, epilepsy, polyimide, strips and grids, electrochemistry, thin-film, neural electrodes

## Abstract

Subdural electrode arrays are used for monitoring cortical activity and functional brain mapping in patients with seizures. Until recently, the only commercially available arrays were silicone-based, whose thickness and lack of conformability could impact their performance. We designed, characterized, manufactured, and obtained FDA clearance for 29-day clinical use (510(k) K192764) of a new thin-film polyimide-based electrode array. This study describes the electrochemical characterization undertaken to evaluate the quality and reliability of electrical signal recordings and stimulation of these new arrays. Two testing paradigms were performed: a short-term active soak with electrical stimulation and a 29-day passive soak. Before and after each testing paradigm, the arrays were evaluated for their electrical performance using Electrochemical Impedance Spectroscopy (EIS), Cyclic Voltammetry (CV) and Voltage Transients (VT). In all tests, the impedance remained within an acceptable range across all frequencies. The different CV curves showed no significant changes in shape or area, which is indicative of stable electrode material. The electrode polarization remained within appropriate limits to avoid hydrolysis.

## 1. Introduction

Subdural strip and grid electrodes are essential tools in the pre-surgical evaluation of patients with drug-resistant epilepsy (DRE). The electrodes are used (1) to record and monitor brain activity with the goal of precisely identifying abnormally seizing tissue and (2) to deliver electrical stimulation for functional brain mapping with the goal of identifying the relationship between eloquent cortical areas (e.g., speech, movement, vision) and seizure onset zones and propagation [1,2,3,4,5]. Recordings are conducted for various lengths of time, ranging from minutes (e.g., intraoperative monitoring during tissue resection) to up to 29 days (e.g., when monitoring patients in the epilepsy monitoring units, EMU) [1,2,3,4,5]. Neural activity is recorded over a wide frequency bandwidth, with the typical bandwidth of interest for epilepsy monitoring being between 1–500 Hz [6,7]. For electrical stimulation used for brain mapping, the stimulation parameters, i.e., the amplitude, pulse width, pulse frequency and duration, are established for each patient and brain area being stimulated [8,9,10,11,12,13]. For safety, the recommended limit on the charge density of a stimulation pulse is 30 µC/cm^2^ for electrodes having a geometric surface area of 0.06 cm^2^, based on histological evaluation of tissue damage in the brains of animal models [13,14,15,16,17,18].

While silicone-based subdural electrodes have been in the market for many years (first FDA clearance in 1985), they are thick (>0.5 mm) and not flexible enough to conform to brain convolutions. Several studies have shown that thickness and lack of conformability can impact the recording quality, put pressure on the brain, creating a ‘mass effect’, and ultimately increase the potential for post-operative complications [19,20,21,22,23,24,25]. To overcome these issues, companies and academic research laboratories have focused on new materials, such as polymers, that can be more conformable, flexible (e.g., polyimide), and amenable to thin sheet (e.g., thinner than 0.1 mm) manufacturing processes. In this study, we describe a 0.08 mm thick electrode array made of polyimide with platinum (Pt) contacts [26].

The ability of subdural electrodes to perform their main functions, i.e., monitoring/recording brain signals with high fidelity over a wide frequency band and providing electrical stimulation to brain tissue within safe limits, depends on the electrode metal contact interaction with the tissue. Characterization of the electrode/tissue interface is a critical step in predicting electrode performance and detecting any potential failures that can impact electrode performance and safety. The typical methods employed for characterization include electrochemical impedance spectroscopy (EIS), cyclic voltammetry (CV) and voltage transient measurements (VT) [27]. EIS evaluates the recording capabilities of the electrodes. CV evaluates electrode contact materials like Pt and characterizes the potential oxidative or reductive reactions that take place at the interface between the contact and the brain. VT estimates the maximum charge that can be injected in a current-controlled stimulation pulse; this is necessary during stimulation for brain mapping to ensure that stimulation limits are below the safety limits for clinical use [13,14,15,16,17,18].

Given the importance of electrochemical characterization for electrode performance and safety, this study describes the results of electrochemical testing of a new, commercially available, thin-film polyimide subdural electrode intended for temporary use (less than 30-days).

## 2. Materials and Methods

### 2.1. Electrodes

**Figure 1:**
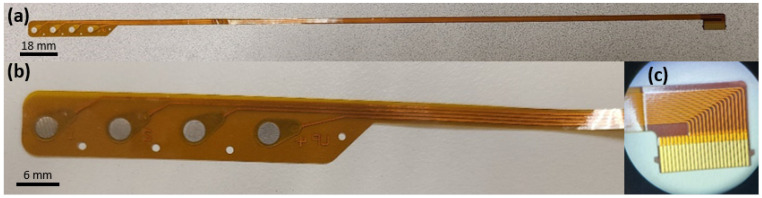
(**a**) Picture of a 1 × 4 strip, tail, and connector. (**b**) High magnification of the electrode contacts. (**c**) 5× magnification of the connector.

Subdural electrode arrays were manufactured by NeuroOne Medical Technologies Corporation (Eden Prairie, MN, USA) as strips (e.g., 1 × 4, Figure 1) or grids (e.g., 4 × 4). The electrodes were made of polyimide thin-film substrate (0.08 mm) and high purity Pt as the electrode contact material [26]. The contacts are 3 mm in diameter and spaced 10 mm from center to center. From an electrochemical standpoint, the geometry and construction of the contacts are identical to any other model of the cortical array. Consequently, the testing presented in this study was performed on 1 × 4 grids, but the results are applicable to all models. The electrode connector, shown in Figure 1c, is an off-the-shelf flex circuit connector with a single-tail design and a proprietary cable assembly.

### 2.2. Testing Paradigms

**Figure 2:**
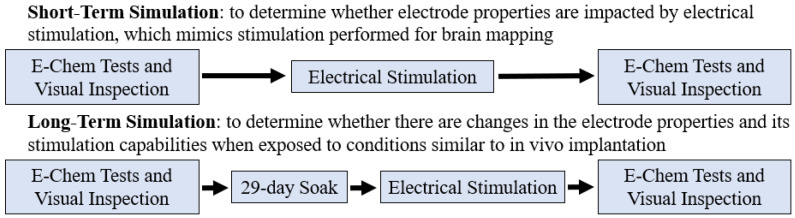
Schematic of testing paradigms. E-Chem tests consisted of EIS, CV, and VT.

The devices underwent two testing paradigms, a short-term electrochemical test and long-term soak followed by electrochemical testing (Figure 2). The short-term test was designed to determine whether the electrode properties are impacted by delivering the types of electrical stimulation required for brain mapping. The test consisted of electrical pulses delivered at parameters similar to functional brain mapping in the clinic [13,14,15,16,17,18]. Stimulation was performed using a Cosman G4 pulse generator (Boston Scientific, Marlborough, MA, USA). Constant-current, charge-balanced stimulation pulses at 10 mA with a pulse width of 0.5 ms and a frequency of 50 Hz were run for a total of 125 s to deliver a total of 31,250 µC of charge on each contact pair of 1 × 4 grids; this represents the worst-case foreseeable charge level during clinical use of 24,210 µC [13,16,17] plus a safety factor of 1.3. All electrochemical tests were run within poly extraction vials containing 1× PBS solution (Antylia Scientific, Vernon Hills, IL, USA) at room temperature.

The long-term test was designed to determine whether there are changes in the electrode properties and its stimulation capabilities when exposed to phosphate buffered saline (PBS) for the duration of the longest authorized implantation period. The test consisted of electrical stimulation to establish baseline values followed by a 29-day passive soak, during which the devices were immersed in 1× PBS at body temperature (37 ± 3 °C). The test ended with electrical stimulation to simulate functional brain mapping. The results of pre- and post-testing were compared to evaluate changes in material performance.

To evaluate device performance over time, prior to and following each testing paradigm, devices were characterized using visual inspection and three types of electrochemical assessments (E-Chem tests, Figure 2): EIS, CV, and VT [27]. 12 contacts (3 arrays of 1 × 4 contacts) were evaluated for each testing paradigm.

### 2.3. Electrochemical Impedance Spectroscopy

The electrochemical tests were conducted with a Biologic SP-200 potentiostat (Biologic, Knoxville, TN, USA) controlled by Biologic EC-Lab software (Biologic, Knoxville, TN, USA) on a Windows-compatible computer.

EIS was performed in a three-electrode cell configuration with an Ag/AgCl (Antylia Scientific, Vernon Hills, IL, USA) reference electrode, a high-area platinized titanium counter electrode (Salmue, China), and the electrode to be analyzed as the working electrode, all immersed in 1× PBS at room temperature. EIS was measured in potential control mode with a sinus amplitude of Va = 10 mV between frequencies of 1Hz to 100 kHz with 12 frequencies per decade and 20 measurements per frequency [28,29,30]. Data analysis was performed to determine the average impedance magnitude and phase at 100 Hz and 1 kHz. The average thermal noise introduced by the electrochemical interface was calculated as the sum of thermal noise at each frequency bin within the bandwidth of interest for epilepsy monitoring (1–500 Hz) [14,15,18]. Frequency bins were created within 1–500 Hz around each frequency at which impedance was measured, and thermal noise was calculated for each bin. The minimum and maximum bandwidth, which is the total range of frequencies that the signal occupies, were calculated for each frequency bin. The local noise within each bin, V_rms_, was calculated using the equation V_rms_ = √4kTBR, where k was the Boltzmann constant, T was the temperature (37 °C), B was the difference between the minimum and maximum bin bandwidth, and R was the impedance magnitude. The thermal noise for the 1–500 Hz bandwidth was then calculated as the square root of the sum of thermal noise power (V_rms_^2^) of each bin.

### 2.4. Cyclic Voltammetry

The devices were designed to record electrical signals with high fidelity as well as deliver electrical stimulation for brain mapping. Pt was chosen as the contact material for this interface due to its high electrical conductivity, inert nature, and corrosion resistance [31,32,33]. To demonstrate that Pt is the only electrochemically active material on the device, CV was used to evaluate the behavior of the device as a function of electrical potential. This experiment also characterized the electrochemically active material within the device to evaluate whether nonreversible chemical reactions occur within clinically relevant potentials [34]. CV was performed in the same three-electrode cell setup described previously. The voltammograms of the electrodes were scanned between potential limits of −0.8 V to 1.2 V at a scan rate of 50 mV/s [35]; these limits were established through a wide voltaic sweep from −2 V to 2 V on a separate device to determine the voltages at which large-scale runaway reactions would begin to occur, also known as the water window. Data analysis was performed to determine the Faradaic peaks on the plot and their accompanying reactionary sequence. The average charge storage capacity, defined as the maximum charge available for a single electrical pulse, was calculated by integrating the entire voltammogram and dividing it by the scan rate and working surface area.

### 2.5. Voltage Transients

VT measurements demonstrated the polarization of the device during typical stimulation parameters and were used to determine whether over-polarization occurred. VT was performed in a three-electrode cell configuration with an Ag/AgCl (Antylia Scientific, Vernon Hills, IL, USA) reference electrode, and the electrode pair to be analyzed as the counter and working electrodes, all immersed in 1× PBS at room temperature. Constant-current, cathodic-first, charge-balanced stimulation was performed at 4 mA and a 10 µs interphase delay. VTs with a pulse width of 200 µs were run before and after testing to ensure that baseline electrical pulses were able to be applied without over-polarizing the electrodes. VTs with a pulse width of 535 µs were run after simulation to determine if the application of electrical pulses at the clinically relevant charge density of 30 µC/cm^2^ would over-polarize the electrodes. Three sets of pulses were run during each trial [34]. Data analysis was performed to determine the maximum change in voltage during polarization. This value was taken from the potential at the end of the current pulse, which represented the highest amount of polarization that the electrode would be subjected to during testing.

### 2.6. Visual Inspection

Visual inspection of all contacts was performed before and after each testing paradigm using an Olympus BX60MF microscope (Olympus, Shinjuku City, Tokyo, Japan). The test assessed the presence of degradation and corrosion qualitatively.

### 2.7. Statistical Analysis

Statistically significant differences between pre- and post-electrochemical testing values for each parameter were tested using paired one-tail Student’s *t*-test. Statistically significant differences between pre- and post-electrochemical testing variance values for EIS and thermal noise were tested using paired two-tail Student’s F-test. A *p*-value less than 0.05 was considered statistically significant.

## 3. Results

### 3.1. Short-Term Electrochemical Testing

**Figure 3:**
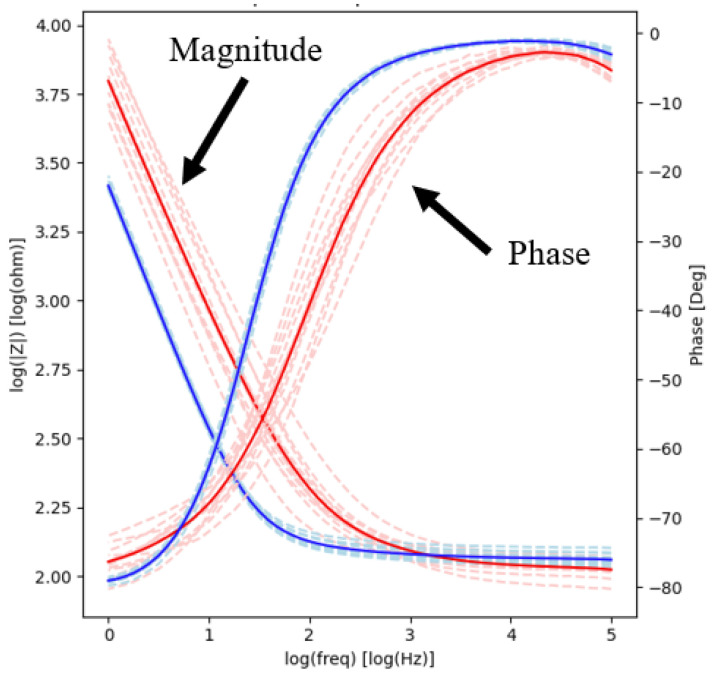
EIS plots before and after short-term electrochemical testing. Impedance magnitude (left Y scale) and impedance phase (right Y scale) are plotted as a function of frequency. The red and blue lines are impedance magnitude measurements made before and after the electrochemical testing, respectively. The dashed lines represent all data recorded, and the solid lines are the averages of the respective category.

The electrochemical impedance magnitude and phase spectrograms for all contacts before and after short-term stimulation are illustrated in Figure 3. Prior to stimulation, the impedance magnitude as a function of frequency showed a linear negative slope from 1 Hz to 3 kHz, with values ranging from 150 Ω to 10 kΩ. From 3 kHz to 100 kHz, the slope of the magnitude was near 0, and values asymptotically approached 100 Ω. After stimulation, the impedance magnitude showed a similar trend, i.e., linear negative slope from 1 Hz to 3 kHz, with values ranging from 150 Ω to 2.5 kΩ, and asymptotically approached 100 Ω from 3 kHz to 100 kHz. Similar behavior was observed for the phase. Prior to stimulation, the phase angle was −80° between 1 Hz to 10 Hz and then showed a linear positive slope from 100 Hz to 10 kHz, with values ranging from −80° to 0°. From 10 kHz to 100 kHz, the slope of the phase was near 0 and values asymptotically approached 0°. After short-term stimulation, the phase displayed similar trends, with the phase angle showing a linear positive slope from 1 Hz to 1 kHz.

The average impedance magnitude and phase at 100 Hz and 1 kHz decreased post-stimulation (Table 1). The variability of the measurements also decreased (F-tests performed on the variance of the impedance data revealed that they were statistically different; *p* < 0.05). The average thermal noise within the 1–500 Hz frequency band was 0.445 ± 0.0311 µV_rms_ and 0.275 ± 0.0244 µV_rms_ before and after stimulation, respectively (*p* < 0.05). This indicates that stimulation does not increase thermal noise and suggests that the quality of the recordings will not degrade after stimulation.

**Figure 4:**
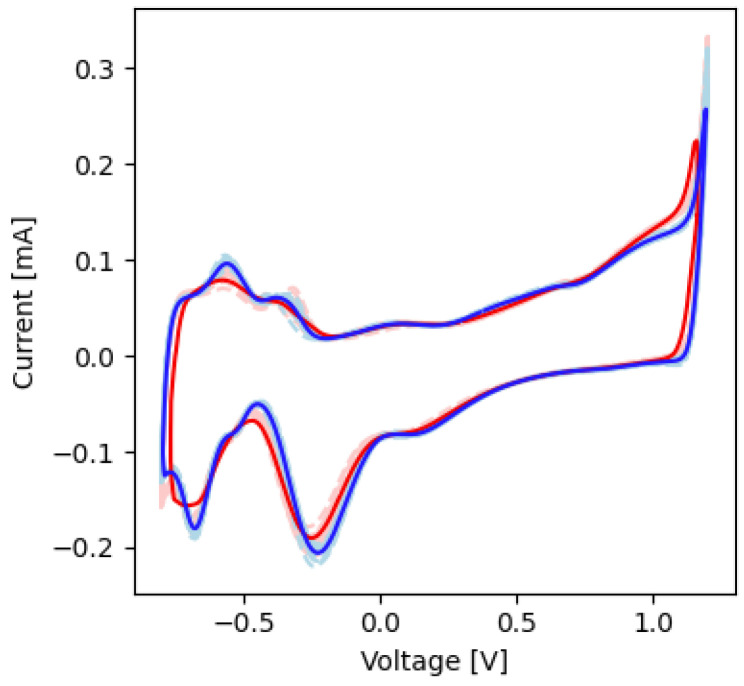
CV plots before and after short-term electrochemical testing. The dashed red and blue lines represent measurements made before and after electrical stimulation, respectively. The solid red and blue lines are the averages of the values before and after electrical stimulation, respectively.

In the CV experiments, the voltammograms showed faradaic peaks corresponding to Pt-O and Pt-H electrochemical reactions (Figure 4). The voltages and current magnitudes of these peaks are shown in Table 2. The average charge storage capacity for these experiments before and after electrical stimulation was 745 ± 13.6 and 747 ± 11.9 µC/cm^2^, respectively (*p* > 0.05).

**Figure 5:**
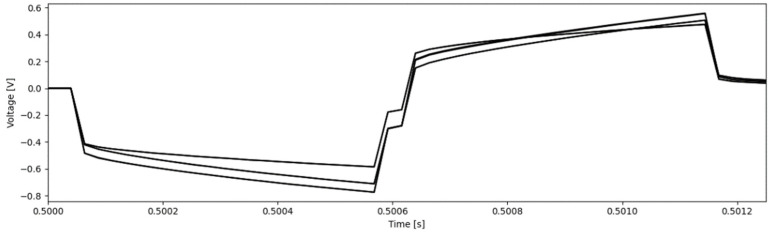
VT plot of one set of pulses at a charge density of 30 µC/cm^2^ after short-term electrochemical testing.

The VT measurements at 200 µs pulse widths showed that the electrodes reached a maximum polarization of 0.380 ± 0.044 V and 0.392 ± 0.041 V before and after electrical stimulation, respectively (*p* > 0.05). The VT measurement at 535 µs pulse width reached a maximum polarization of 0.658 ± 0.100 V and is shown in Figure 5; these values were within the clinically safe limits of −0.8 to 1.2 V [34].

**Figure 6:**
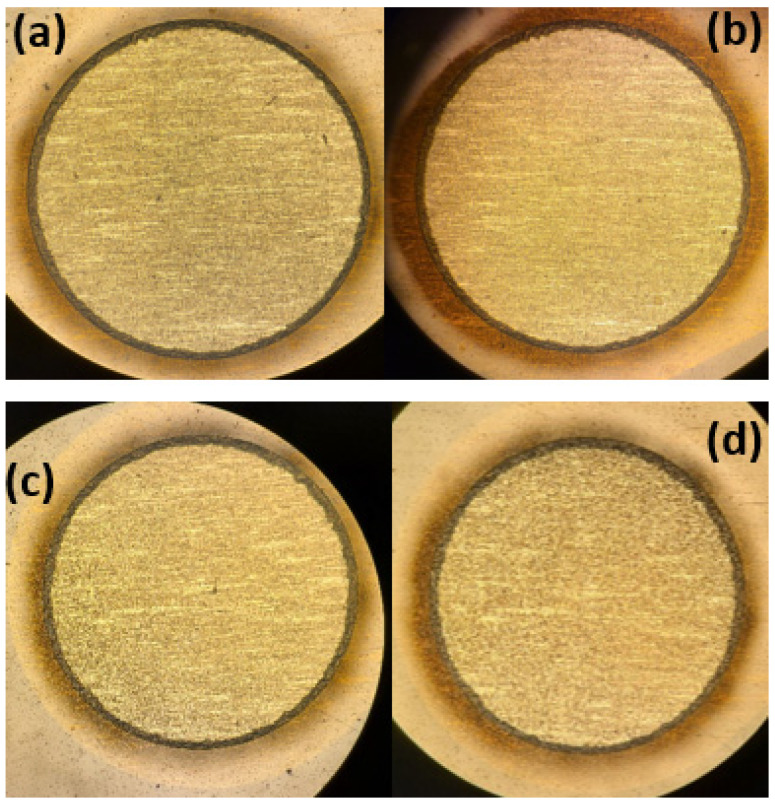
Visual inspection of electrodes. Selected images of electrode contacts pre- and post-electrochemical testing on the left (**a**,**c**) and right (**b**,**d**), respectively. The top images (**a**,**b**) are from short-term electrochemical testing, and the bottom images (**c**,**d**) are from long-term electrochemical testing. The contact is 3 mm, and the magnification is 5×.

Visual inspection of all contacts revealed no corrosion, degradation, or metal delamination (example in Figure 6a,b).

### 3.2. Long-Term Electrochemical Testing

**Figure 7:**
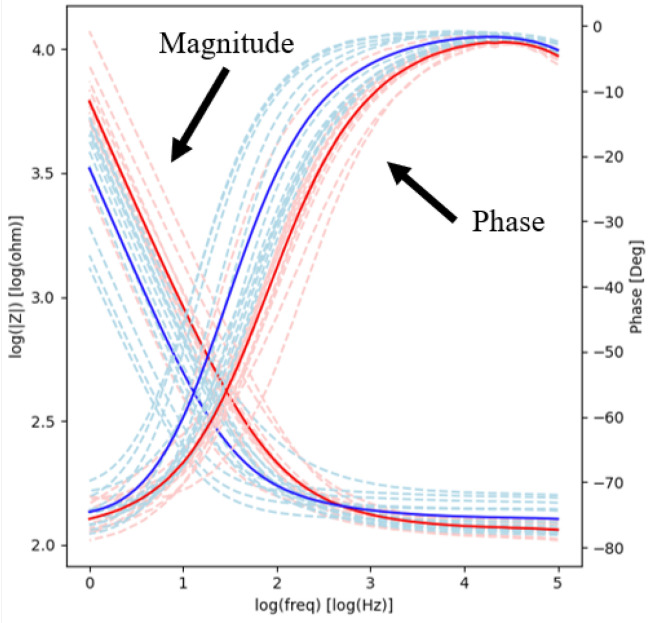
EIS plots before and after long-term electrochemical testing. Impedance magnitude (left Y scale) and impedance phase (right Y scale) are plotted as a function of frequency. The red and blue lines are impedance magnitude and phase measurements made before and after soak and electrical stimulation, respectively. The dashed lines represent all data recorded, and the solid lines are the averages of the respective category.

The electrochemical impedance magnitude and phase spectrograms for all contacts before and after 29-day soaking are illustrated in Figure 7. Before soaking, the impedance magnitude showed a linear negative slope from 1 Hz to 100 Hz, with values ranging from approximately 125 Ω to 5.6 kΩ. From 100 Hz to 100 kHz, the slope of the magnitude was near 0 and asymptotically approached 100 Ω. After soaking (long-term electrochemical testing), the magnitude of impedance showed a linear negative slope from 1 Hz to 100 Hz, with values ranging from approximately 125 Ω to 3.1 kΩ, and asymptotically approached 100 Ω from 100 Hz to 100 kHz. Prior to soaking, the phase angle showed a positive linear slope from −75° to 0° between 1 Hz to 10 kHz and asymptotically approached 0° at higher frequencies. After soaking, the phase angle had a positive linear slope from −75° to 0° between 1 Hz to 300 Hz and asymptotically approached 0° from 300 Hz to 100 kHz. The average impedance magnitude and phase at 100 Hz and 1 kHz decreased post-stimulation (Table 3). The variability of the measurements also decreased (F-tests performed on the variance of the impedance data revealed that they were statistically different; *p* < 0.05). The average thermal noise within the 1–500 Hz frequency band was 0.361 ± 0.0271 µV_rms_ and 0.114 ± 0.0138 µV_rms_ before and after stimulation, respectively (*p* < 0.05).

**Figure 8:**
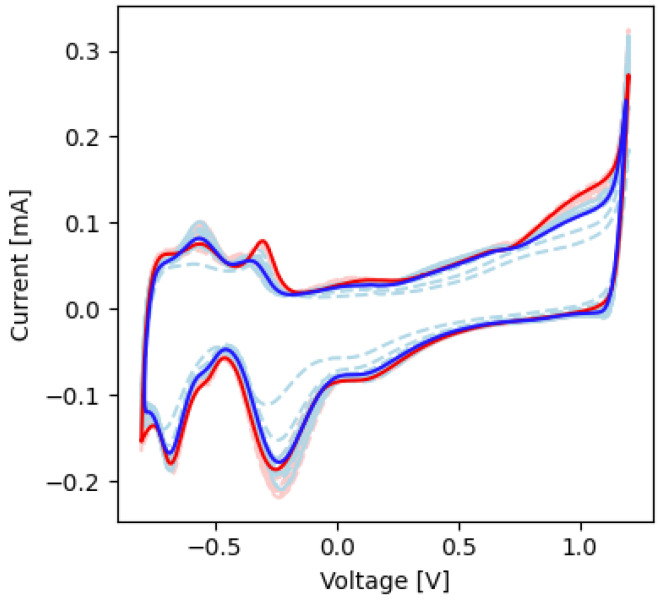
CV plots before and after long-term electrochemical testing. The dashed red and blue lines represent measurements made before and after soak an eletrical stimulation, respectively. The solid red and blue lines are the averages of the values before and after soak and electrical stimulation, respectively, respectively.

The voltammograms showed faradaic peaks before and after stimulation that were indicative of Pt-O and Pt-H electrochemical reactions (Figure 8). The voltages and current magnitudes of these peaks are shown in Table 4. The average charge storage capacity before and after soaking was 741 ± 17.2 and 681 ± 22.3 µC/cm^2^, respectively (*p* < 0.05).

**Figure 9:**
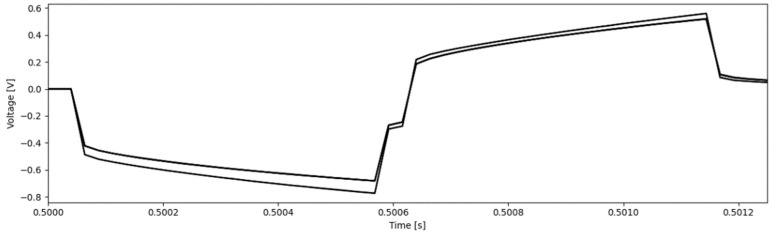
VT plot of one set of pulses at a charge density of 30 µC/cm^2^ after long-term electrochemical testing.

The voltage transient measurements at 200 µs pulse width showed that the maximum change in voltage during polarization at 4 mA was 0.405 ± 0.030 V and 0.411 ± 0.036 V before and after soak and electrical stimulation, respectively (*p* > 0.05). The VT measurement at 535 µs pulse width reached a maximum polarization of 0.668 ± 0.075 V (Figure 9); this indicates that the electrodes did not over-polarize past the clinically safe limits of −0.8 and 1.2 V.

Optical analysis of all contacts revealed no visual contact corrosion, degradation, or metal delamination due to electrical stimulation (example in Figure 6c,d).

## 4. Discussion

This study tested the electrochemical properties of the first thin-film-based cortical electrode cleared by the FDA for less than 30-day clinical use. The characterization techniques presented here represent the standard characterization and have been extensively used in academic settings [27,28,30,37], providing a large body of literature to quantitively compare the characteristics and performance of commercially available subdural electrodes. Characteristics such as signal-to-noise ratio and the ability of the device to safely apply electrical stimulation are important to clinicians who intend to use these devices [30,32,34,38]. In this study, the electrochemical properties of the characterized electrodes demonstrate that the impedance of the contacts falls within ranges known to provide low noise recordings (up to 1 kΩ) [28,30] at the frequencies of interest for epilepsy monitoring [8,12]. Similarly, the results demonstrate that the contact material has adequate properties to deliver electrical pulses and does not exhibit degradation after the expected maximum levels of stimulation during the intended application [34].

The key metrics of interest in the electrochemical characterization of the cortical electrodes were the ability to record neural signals in the bandwidth relevant to epilepsy monitoring, the ability to apply electrical stimulation without material degradation, and the ability to inject enough electrical charge as expected in cases of most exhaustive brain mapping. The test protocols written and implemented for each of these metrics were electrochemical impedance spectroscopy, cyclic voltammetry, and voltage transients of devices subjected to passive soak and electrical stimulation [27]. In addition, a visual inspection to evaluate the material surface was carried out for each electrode. The EIS measurements (Figure 3 and Figure 7) showed the expected frequency-dependent behavior of an electrochemical interface due to the capacitive component [29]: low impedance magnitude at phase near zero degrees for high frequency, high impedance magnitude with phase near ninety degrees for low frequencies, and a logarithmic dependency of magnitude and phase on frequency. At high frequencies (e.g., >3 kHz), where the impedance is dominated by the impedance of the electrode itself [29,39], the impedance magnitude plateaued near 100 Ω. At other characteristic frequencies like 100 Hz and 1 kHz, the impedance is similar or lower than the impedance of other reported electrode contacts of similar dimensions; these other contacts have performance that can range in the order of kΩ, despite them not been subjected to special surface treatments or coatings intended to lower impedance [15,30]. The low average thermal noise further showed that the electrodes are capable of high-fidelity signal recording. Post-stimulation recordings indicated that the impedance magnitude, phase, and thermal noise decreased. The variability of these measurements also decreased (statistically significant differences in variance for magnitude and phase) relative to pre-stimulation values (Table 1 and Table 3). It is likely that the polarization of the device removed any residual impurities on the electrochemical interface of the contacts due to cyclic voltammetry and stimulation, which resulted in more precise readings [40]. The removal of these impurities would have enlarged the electrochemically active interface, which would result in lower impedance.

With regards to the electrode’s ability to safely perform electrical stimulation, the voltammograms of all contacts tested had faradaic peaks indicative of Pt metals with anodic and cathodic peaks at voltages characteristic of Pt-O and Pt-H reactions [32,33]. As stated previously, Pt is a common electrochemically inert material widely used in neural devices due to its high corrosion resistance, biocompatibility, and electrical properties [32,33,34]. The CV curves (Figure 4 and Figure 8) are characteristic of pure Pt, with no additional peaks associated to alternative materials observed [32,33]. The charge storage capacity of the devices was higher than the clinically recommended charge application limit of 30 µC/cm^2^, meaning that no charge was lost during stimulation [36]. Similarly, the results from the voltage transient measurements show that the electrode was not over-polarized when stimulated up to the recommended charge limit of 30 µC/cm^2^ (Table 2 and Table 4, Figure 5 and Figure 9) [13,14,15,16,17,18,35,41]; this helps confirm the devices’ ability to operate in the range of clinically relevant charge densities of 0–30 µC/cm^2^. Lastly, the lack of changes in the quality and surface appearance of the electrode contacts, as assessed by visual inspections (Figure 6) provides a demonstration that the Pt films are not being degraded or corroded.

The electrodes characterized as part of this study showed statistically significant differences in the EIS and CV results before and after soak and/or electrical stimulation. While all ranges measured were demonstrated to be safe and functional, the root of this variability is of particular interest. The EIS distribution of working electrode contacts in similar designs is often very tight [42,43]. Both measurements are particularly sensitive to the surface area [44], and it is hypothesized that the variation within the allowable tolerance of the contact diameter, as well as the variation of the surface roughness of the Pt film, can account for the variability in the measurements. As stated previously, the polarization of the devices during the presence of electrical signals can lead to changes on the electrochemical surface, such as the removal of impurities; these events alter the electrochemically active surface area, which results in shifts in the EIS and CV data. All data before and after soak and electrical stimulation were within clinically safe ranges, despite their statistical significance from one another.

It is important to highlight the context within which these experiments were carried out, which was to provide necessary data for the FDA to clear these devices for commercial use for recording brain activity and performing brain stimulation for periods of less than 30 days; these results demonstrated baseline reliability and electrical safety of the devices and validated that stimulation and electrical detection capabilities were maintained for up to 29 days; these results were the basis to set passing criteria and develop test methods performed during design verification of the NeuroOne Evo Cortical electrode. Additional formal testing for biocompatibility [26], sterility, mechanical properties, lot-to-lot variability, shelf life, and other qualities of the device was conducted to gain clearance of this device.

## 5. Conclusions

Electrochemical evaluation is a critical step in the path to the commercialization of implantable medical devices. This study presented the electrochemical characterization of the first thin-film-based cortical electrode cleared by the FDA for commercial use for recording brain activity and brain stimulation for less than 30 days. Techniques including electrochemical impedance spectroscopy, cyclic voltammetry, and voltage transients were used within the context of electrically passive and active simulations to demonstrate the expected performance of this electrode. All measurements performed were consistent with commercially available subdural electrodes reported in previous academic articles, and all tests demonstrated acceptable performance of the device. Currently, this subdural electrode is being successfully used clinically, indicating that the bench top tests were adequate to predict safe and effective electrochemical performance.

## Figures and Tables

**Table 1:** EIS measurements before and after short-term test.

	100 Hz		1 kHz	
	Magnitude (Ω)	Phase (°)	Magnitude (Ω)	Phase (°)
Before	218.72 ± 39.29	−37.22 ± 5.72	133.02 ± 8.33	−10.88 ± 2.78
After	146.83 ± 5.43 *	−20.46 ± 0.74 *	127.32 ± 5.48	−4.29 ± 0.25 *

Average and standard deviation of impedance magnitude and phase before and after short-term stimulation at 100 Hz and 1 kHz. Asterisks indicate statistically significant differences (*p* < 0.05) between pre- and post-values, tested using a paired *t*-test.

**Table 2:** CV faradaic peaks and associated electrochemical reactions before and after short-term electrochemical testing.

Before Stimulation		After Stimulation		Relevant Reaction
Peak Voltage (V)	Peak Current (mA)	Peak Voltage (V)	Peak Current (mA)	
−0.25	−0.18	−0.22	−0.21	Pt-O reduction
−0.70	−0.14	−0.70	−0.17	Pt-H reduction
−0.52	0.08	−0.51	0.10	Pt-H oxidation
−0.45	0.07	−0.45	0.08	Pt-H oxidation

The relevant reaction was determined according to previous studies [36].

**Table 3:** EIS measurements before and after long-term electrochemical testing.

	100 Hz		1 kHz	
	Magnitude (Ω)	Phase (°)	Magnitude (Ω)	Phase (°)
Before	211.23 ± 39.29	−39.20 ± 5.72	124.01 ± 8.33	−11.75 ± 2.78
After	133.81 ± 5.43 *	−16.39 ± 0.74 *	120.39 ± 5.48 *	−3.28 ± 0.25 *

Average and standard deviation of impedance magnitude and phase before and after short-term stimulation at 100 Hz and 1 kHz. Asterisks indicate statistically significant differences (*p* < 0.05) between pre- and post-values, tested using a paired *t*-test.

**Table 4:** CV faradaic peaks and associated electrochemical reactions before and after long-term electrochemical testing.

Before Stimulation		After Stimulation		Relevant Reaction
Peak Voltage (V)	Peak Current (mA)	Peak Voltage (V)	Peak Current (mA)	
−0.25	−0.18	−0.23	−0.17	Pt-O reduction
−0.75	−0.17	−0.76	−0.16	Pt-H reduction
−0.55	0.07	−0.55	0.08	Pt-H oxidation
−0.42	0.08	−0.40	0.06	Pt-H oxidation

The relevant reaction was determined according to previous studies [36].

## Data Availability

All relevant data are included in the manuscript and on file at NeuroOne Medical Technologies Corporation.
